# A Mini-Review on Detection Methods of Microcystins

**DOI:** 10.3390/toxins12100641

**Published:** 2020-10-04

**Authors:** Isaac Yaw Massey, Pian Wu, Jia Wei, Jiayou Luo, Ping Ding, Haiyan Wei, Fei Yang

**Affiliations:** 1 Xiangya School of Public Health, Central South University, Changsha 410078, China; mriymassey@csu.edu.cn (I.Y.M.); wupian@csu.edu.com (P.W.); wjcindy@csu.edu.cn (J.W.); guojianph@csu.edu.cn (J.L.); pingshui@csu.edu.cn (P.D.); 2 Department of Occupational Medicine and Environmental Toxicology, School of Public Health, Nantong University, Nantong 226019, China; 3 School of Public Health, University of South China, Hengyang 421001, China

**Keywords:** detection, microcystins, ELISA, HPLC-MS, biosensor

## Abstract

Cyanobacterial harmful algal blooms (CyanoHABs) produce microcystins (MCs) which are associated with animal and human hepatotoxicity. Over 270 variants of MC exist. MCs have been continually studied due of their toxic consequences. Monitoring water quality to assess the presence of MCs is of utmost importance although it is often difficult because CyanoHABs may generate multiple MC variants, and their low concentration in water. To effectively manage and control these toxins and prevent their health risks, sensitive, fast, and reliable methods capable of detecting MCs are required. This paper aims to review the three main analytical methods used to detect MCs ranging from biological (mouse bioassay), biochemical (protein phosphatase inhibition assay and enzyme linked immunosorbent assay), and chemical (high performance liquid chromatography, liquid chromatography-mass spectrometry, high performance capillary electrophoresis, and gas chromatography), as well as the newly emerging biosensor methods. In addition, the current state of these methods regarding their novel development and usage, as well as merits and limitations are presented. Finally, this paper also provides recommendations and future research directions towards method application and improvement.

## 1. Introduction

Cyanobacterial harmful algal blooms (CyanoHABs) are globally on the increase in both frequency and intensity as a result of eutrophication and climate change [1,2,3]. The most frequently reported CyanoHABs toxins are cyclic heptapeptide hepatotoxins microcystins (MCs) which have attracted worldwide studies. MCs most often found in water and to a lesser extent in desert environments are primarily produced by cyanobacteria species of the genera *Microcystis*, *Anabaena*, *Aphanizomenon*, *Nostoc*, *Cylindrospermopsis,* and *Planktothrix* [2,4,5].

The cyclic heptapeptide hepatotoxins are relatively stable in natural environments and resistant to chemical and physical factors including extreme temperatures, pH changes, sunlight and degradation via non-specific enzymes owing to their cyclic structure [6,7,8]. The common structure of MCs is cyclo-(-D-Ala-L-X-DisoMeAsp-L-Z-Adda-D-isoGlu-Mdha), where X and Z are highly variable amino acids, D-MeAsp is D-erythro-b-methylaspartic acid, Mdha is N-methyldehydroalanine, and Adda is (2S, 3S, 8S, 9S)3-amino-9 methoxy-2,6,8-trimethyl-10-phenyldeca-4, 6-dienoic acid [9,10,11]. More than 270 MC variants have been isolated from CyanoHABs [12]. On the basis of toxicity microcystin-LR (MC-LR) is by far the most potent hepatotoxin among the different variants of MC and has become a global focus [13,14,15]. The International Agency for Research on Cancer classified this toxin as a group 2B carcinogen [16], and the World Health Organization (WHO) recommended a provisional 1 µg/L MC-LR guidelines for drinking water quality [17].

In recent years, MCs production has been reported from all continents especially from tropical and subtropical areas under an extensive variety of environmental conditions [18,19,20,21,22,23,24,25,26]. Human and animal health problems are prone to be associated with chronic exposure of MCs concentration primarily through ingestion and body contact [15]. MCs are potent and specific inhibitors of protein phosphatases 1 (PP1) and protein phosphatases 2A (PP2A) from both mammals and higher plants [27]. This may alter the expression levels of miRNA, induce cytoskeleton disruption, DNA destruction, inflammation, autophagy and apoptosis [14,28,29,30]. Exposure to MCs may severely damage mammalian organs including the liver, intestines, brain, heart, lungs, kidney and reproductive system. In addition, through the accumulation of these toxins, plants growth and yield may be threatened [5,15,31]. This may further exhibit moderate or high human health risk and intoxicate other organisms through food transfer.

To effectively manage and control MCs, as well as prevent or minimize their health risks, sensitive, fast and reliable screening methods capable of detecting these toxins are urgently required. Early detection of MCs can help to counteract these deadly toxins, to avoid further posing ecosystem and human health threat. An important consideration in analyzing water samples for MCs is to determine the differences between intracellular and extracellular toxins [32]. To successfully determine the toxins level, there should be cell lysis to release intracellular toxins, mostly by freeze-thawing and ultrasonication bath [33,34,35]. Therefore, the first step towards MCs hazards prevention must contain developing sensitive, fast and reliable screening methods to identify these toxins. Thus, the paper aims to review the analytical and biosensor methods used for MCs detection in terms of their novel development and usage, as well as merits and limitations. The paper also puts forward some directions for future research towards method application and improvement.

## 2. Analytical Methods to Detect Microcystins

### 2.1. Biological Method

#### Mouse Bioassay (MBA)

This method is mainly used to detect MCs in animals with unknown toxins composition (Table 1). Generally, toxins extracts are administered via intraperitoneal injection into mice. The lethal dose LD_50_ by intraperitoneal route ranges from 50 (MC-LR) to 600 (MC-RR) µg/kg while oral LD_50_ is 5000 µg/kg. MBA may also employ microbes, invertebrate and vertebrate animals, cell cultures or plants and plant extracts to detect MCs [36]. MBA was one of the techniques used to investigate the Hartebeespoort dam (South Africa), Malpas dam (New England region of Australia) and Paraná River (Argentina) for MC-LR and MCs toxicity [37,38,39].

The major merit of MBA is that it makes effective use of the whole animal, which is a more realistic approach to detect MCs toxicity. The animal used has the ability to provide natural physiological and biochemical functions to help detect the toxins [36,39,40]. MBA is usually used in a more qualitative way to detect MC variant(s) and toxicity present in water samples. In addition, it can be calibrated against a specific MC variant to generate results in terms of MC toxicity equivalents [33,36,41].

The major limitations of MBA have been identified as lack of providing a realistic way to analyze MCs, lack of sensitivity, and not being suitable for quantification purposes [17,33,38,39]. Besides, due to ethical reasons, MBA is not an appropriate technique for large scale and routine testing of MCs in water samples. The number of mice needed to perform MBA is mostly unfeasible and unacceptable. Moreover, unless a license is obtained, a number of countries do not permit its use. The few accepting countries are limited by animal house facility for rearing the mice for routine experiments [33,39,40]. This has led to the fading of MBA technique.

**Table 1:** Analytical methods to detect microcystins in water.

Source	Analytical Method (Test)	Sample Preparation	Major Species	MC Variant Detected	Amount of Toxin Detected	Reference
Raw and treated waters	HPLC-PDA	C_18_ SPE cartridge	*Microcystis aeruginosa*	MC-RR, MC-LR, MC-LY, MC-LW and MC-LF	0.034–8.880 µg/L	[42]
Lake, river, dam and shoreline	ELISA	-	*Microcystis* sp.	MC-LR and MC-RR	0.2–200 ng/mL	[43]
Water samples	HPLC-UV, CE-UV and LC-MS/MS	-	*Microcystis aeruginosa*	MC-LR, MC-LY, MC-AR, MC-LF, MC-LW and MC-VF		[44]
Water samples	CE	-		MC-LR, M-YR and M-RR		[45]
Lake Suwa	EI-GC/MS	-	*Microcystis aeruginosa*	MC-LR	0.97–8.85 n/mg	[46]
Lakes (Mira, Barrinha de Mira), Rivers (Minhoand, Guadiana) and reservoirs (Crestuma, Torrfio, Carrapatelo, Aguieira, Vale das Bicas)	Mouse bioassay and HPLC	-	*Microcystis aeruginosa, Microcystis wesenbergii, Anabaena flos-aquae* and *Nostoc* sp.	MC-RR, YR, [DAsp3]MC-LR, HilR, [LMeSer7]MC-LR and [Dha7]MC-LR	1.0–7.1 μg/mg	[40]
Freshwater samples	GC/CI-MS and LC	-	-	MC-RR, MC-YR and MC-LR	0.04–80.41 µg/L	[47]
Water samples	CI-ELISA	-	-	MC-LR, MC-RR, MC-YR, MC-LW, MC-LF, dmMC-LR and dmMC-RR	0.02–0.07 ng/mL	[48]
Water samples	CIPPIA and LC-MS/MS	-	-	MC-LR, MC-D-Asp3, MC-RR, MC-LA, MC-LF, MC-LY, MC-LW, and MC-YR	-	[49]
Finnish lakes	PPIA, HPLC-UV and ELISA	-	*Anabaena* sp., *Oscillatoria* sp. and *Microcystis* sp.	MC-LR, [D-Asp^3^]MC-LR, [Dha^7^]MC-LR, MC-RR, [D-Asp^3^]MC-RR, [Dha^7^]MC-RR, [D-Asp^3^, Dha^7^]MC-RR and MC-YR	0.26–2.5 µg/L	[35]
Drinking water	PPIA	-	-	MC-LR	0.1–1 µg/L	[50]
Water sample	HPLC-UV, CE-UV and CE–ESI-MS	SPE C_18_ disks	-	MC-LR and MC-RR	-	[51]
Water samples	HPLC-DAD/UV and CE- UV-VIS	SPE C_18_ cartridges and IAC	-	MC-RR, MC-LR and MC-YR	2.12–968.80 µg/L	[52]
Lake Oubeira	PPIA and MALDI-TOF-MS	Bakerbond SPE cartridge	*Microcystis aeruginosa*	MC-LR, MC-YR, MC-RR and D-MC-LR	3–29,163 µg/L	[53]
Lake Sonachi and Simbi	HPLC-PDA and MALDI-TOF	Sep-Pak Plus tC_18_ cartridge	*Arthrospira fusiformis, A. fusiformis* and *Anabaenopsis abijatae.*	MC-LR, MC-RR, MC-LA and MC-YR	1.6–39.0 ug/g	[54]
Brno reservoir	HPLC and CEC-UV	-	*Microcystis aeruginosa*	MC-RR, MC-YR and MC-LR	3.6–253.5 µg/L	[55]
Water samples from tap water, River, Lake and swimming pool	IC-ELISA and HPLC	-	-	MC-LR and MC-RR	0.01–5.1 µg/L	[56]
Lake (Xihai, Nanhai, Nanhai, Qianhai, Beihai and golf course)	IC-ELISA and HPLC	-	-	MC-LR, MC-RR and MC-YR	0.1–10 µg/L	[57]
Hartbeespoort dam	ELISA, PPIA and Mouse bioassay	-	*Microcystis aeruginosa* and *Plantothrix* sp.	MC-LR	0.001–86.083 mg/L	[37]
Water samples	pCEC-UV	SPE	-	MC-LR, MC-YR and MC-RR	0.10–0.16 µg/L	[58]
Lake Kavada	ELISA and HPLC-PDA	C_18_ SPE cartridges	*Microcystis aeruginosa, Synechococcus, Phormidium limosum, Phormidium formosa* and *Planktothrix limnetica*	MC-LR, RR, LA, LW and LF	0.5–98.9 µg/L	[59]
Tap water	CZE and MEKC	C_18_ (octadecyl) silica SPE column	-	MC-RR, MC-YR and MC-LR	0.82–4.81 µg/L	[60]
Lakes (Nyabikere Crater, Nkuruba Crater, Nkugute Crate, George, Edward, Mburo, Nabugabo, Victoria), Pond and Swamp	HPLC-DAD, LC-MS/MS and MALDI-TOF MS	-	*Anabaena* and *Microcystis*	[MeAsp3, Mdha7]-MC-RR, [Asp 3]-MC-RY and [MeAsp3]-MC-RY	-	[61]
Water samples collected from different sites in Brazil	HPLC-PDA- ESI-MS/MS and UV spectroscopy	-	*Microcystis* spp.	MC-LR, [D-Asp^3^]-MC-LR, [Asp^3^]-MC-LR, MC-RR, [Dha^7^]-MC-LR, MC-LF, MC-LW and [D-Asp^3^, EtAdda^5^]-MC-LH	-	[62]
Water samples	Natural PP2A, recombinant PP2A and recombinant PP1	-	-	MC-LR, MC-YR and MCRR	8–98 pM	[63]
River Ponjavica	HPLC-PDA	HLB, Sep-Pak	*Microcystis aeruginosa*	MC-LR	1.5 µg/L	[64]
Lake Marathonas	LC-ESI-MS/MS and PPIA	-	*Microcystis* sp.	MC-LA, MC-YR, MC-LR and MC-RR	-	[65]
Manjalar Dam	HPLC and GC-MS	-	*Microcystis aeruginosa*	MC-LR and [D-Asp^3^] MC-LR	-	[66]
Tai lake	GC-MS and LC-MS	-	-	MC-LR and MC-RR	0.56–6.7 µg/L	[67]
Water samples	PP2A_Rec_, PP1_Rec_ and PP2A_Wild_	-	-	MC-LR, MC-RR, MC-dmLR, MC-YR, MC-LY, MC-LW and MC-LF	0.5–3.1 µg/L	[68]
Water samples	HPLC-UV	-	-	MC-LR	0.02 µg/L	[69]
Water samples	Biosensor based on the inhibition of recombinant PP1α	-	-	MC-LR	0.93–40.32 µg/L	[70]
Water samples	HPLC-UV	MSPE	-	MC-LR	0.25–250 µg/L	[71]
Water samples	HPLC	MSPE	-	MC-LR	0.025 µg/L	[72]
Water samples	LC-MS/MS	MSPE	-	MC-LR, MC-RR, MC-YR, MC-LA, MC-LY, MC-LF and MC-LW	0.03–0.61 µg/L	[73]
Dau Tieng Reservoir	Reverse phase HPLC-UV-PDA and UV spectroscopy	-	*Microcystis aeruginosa*	MC-RR, MC-LR and MC-LY	39–2129 µg/g/dw	[74]
Green Lake	LC-HRMS, LC-HRMS/MS and HPLC-DAD	BuOH	*Microcystis aeruginosa*	MC-FR, MC-YR, MC-LR and MC-MhtyR	-	[75]
River Nile	ELISA Kit and HPLC	-	*Microcystis aeruginosa*	MC-RR and MC-LR	1.2–4.5 µg/L	[76]
Lake Tana	ELISA and HPLC-DAD	-	*Microcystis aeruginosa*	MC-LR, MC-RR and MC-YR	0.02–2.65 µg/L	[77]
Lakes	LC-HESI-MS/MS	On-line-SPE	-	MC-RR, MC-YR, MC-LR, MC-LY, MC-LW, and MC-LF	0.029–36 µg/L	[78]
Water sample	IC-ELISA	-	-	MC-LR	0.01–1.63 µg/L	[79]
River, lake and tap water samples	IC-ELISA	-	-	MC-LR, MC-RR, MC-YR, MC-WR, MC-LA, MC-LF, MC-LY, and MC-LW	0.16 µg/L	[80]
Paraná river	HPLC-PDA and Mouse bioassay	-	*Microcystis aeruginosa*	MC-LR, RR and [D-Leu1] MC-LR	0.09–37.7 µg/L	[39]
Water samples	PPIA and LC-MS/MS	-	*Microcystis* spp. and *Anabaena* spp.	MC-LR, MC-LA, MC-RR and -LF	0.20–50 µg/L	[81]
Water samples	ELISA and HPLC-PDA	-	*Microcystis aeruginosa*	MC-LA, MC-YR, MC-LY, MC-LF, MC-RR and MC-LR	0.043–13.5 µg/L	[82]
Helong reservoir and Tianlu Lake	TOF-MS	-	-	MC-LR and MC-RR	0.1–9.1 µg/L	[83]
Lake Garda	LC-MS	-	*Planktothrix rubescens*	MC-RRdm, MC-LRdm, MC-HtyrRdm, MC-RR and MC-LR	20–210 ng/L	[84]
Tai lake	GC-MS and LC-MS	-	-	MC-LR, MC-RR, MC-YR, MC-LY, MC-LA and MC-WR	0.02–2.30 µg/L	[85]
Lagoons	ELISA and LC-MS/MS	SPE C_18_ columns	-	MC-LR	0.04–0.75 ng/L	[86]
Water samples	Noncompetitive ELISA, HPLC and LC-MS	-	-	MC-LR, MC-dmLR, MC-RR, MC-dmRR, MC-YR, MC-LA, MC-LY, MC-LF, MC-LW and MC-WR	<0.06–0.21 µg/L	[87]
Colorado River water and California State Project water	ELISA, LC-MS/MS and PPIA	-	*Microcystis* spp	MC-LR, MC-LA, MC-YR, MC-RR, MC-LF, MC-LW, MC-LY and MCdmLR	<0.1–5 µg/L	[88]
Water samples	HPLC-UV	MSPE	-	MC-LR and MC-RR	0.001 µg/L	[89]
Water samples	LC-QTtoF HRMS	On-line SPE	-	MC-LR, MC-YR, MC-RR, MC-HtyR, MC-HilR, MC-WR, MC-LW, MC-LA, MC-LF, MC-LY, Dha^7^-LR, Dha^7^-RR, Leu^1^-MC-Met(O)R and Leu^1^-MC-LY	0.004–0.01 µg/L	[34]
Natural lake	HPLC-PDA	-	*Microcystis aeruginosa*	MC-WR, MC-RR, MC-DM-WR and MC-YR	-	[90]
Anzali wetland	HPLC-UV	C_18_ SPE cartridges	*Anabaena*	MC-LR	0.18–3.02 µg/L	[91]
Freshwater pond	HPLC-ESI-MS	-	*Microcystis* sp.	MC-LR, MC-RR and MC-LY	-	[92]
Water samples	Adda ELISA, multi-hapten ELISA, NMR spectroscopy, LC-HRMS and LC–MS ^2^	-	*Microcystis* sp.	MC-LR and [D-Leu^1^]MC-LR	24.8–124 ng/g	[93]
Porce II and Riogrande II water reservoirs	HPLC/MS	C_18_ cartridges (CNWBOND HC-C18)	*Microcystis aeruginosa*	MC-LR	124–5729 µg/L	[94]
Water samples (Dongwazi Lake, drinking bottled water from supermarket and tap water)	IC-ELISA-MscFv7-scFv	-	-	MC-LR, MC-RR and MC-YR	0.471- 0.548 µg/L	[95]
Lake Taihu	UHPLC-MS/MS	On-line SPE	-	MC-LR, MC-RR, MC-LY, MC-LW, MC-YR, MC-WR, MC-LF and MC-LA	0.1–3.1 μg/kg	[96]
Hartbeespoort Dam and crocodile farm’s breeding dam	Norwegian ELISA, ELISA kit and LC-HRMS	-	-	MC-LR, MC-RR and MC-YR	0.01–368.79 µg/L	[97]
Water samples	IC-ELISA-PAbs and scFv	-	-	MC-LR, MC-RR, MC-WR and MC-YR,	0.44–1.36 µg/L	[98]
Reservoirs and artificial ponds in Okinawa prefecture	^l^H NMR spectrometry, LC-MS and PP2A	-	*Microcystis aeruginosa*	MC-LR, MC-RR, MC-LA, MC-FR and MC-WR	-	[99]
Saline water	UHPLC-MS/MS and UHPLC-DAD	SALLE	-	MC-RR, MC-YR, MC-LR, MC-WR, MC-LA, MC-LY, MC-LW and MC-LF	0.02–3.4 µg/L	[100]
Lake samples	UHPLC-HRMS	On-line SPE	-	MC-RR, [Asp^3^]MC-RR, MC-YR, MC-HtyR, MC-LR, [Asp^3^]MC-LR, MC-HilR, MC-WR, MC-LA, MC-LY, MC-LW and MC-LF	8–53 ng/L	[101]
Michigan lakes	LC-MS/MS and Adda-ELISA	SPE	-	MC-RR, MC-LA, MC-LR, MC-RR, [D-Asp^3^]MC-LR, MC-YR, MC-HilR, MC-WR, [D-Asp^3^]MC-RR, MC-HtyR MC-LY, MC-LW and MC-LF	0.6–3.8 ng/L	[102]
Lake Uluabat	LC-MS/MS, LC-UV-MS, LC-HRMS and ELISA	-	*Microcystis* spp.	MC-LR, MC-RR, MC-LA, MC-LY, MC-LW, MC-LF, MC-YR, MC-WR, MC-HtyR, [D-Asp ^3^ ]MC-LR, [Dha^7^ ]MC-LR, MC-HilR, [D-Asp^3^ ]MC-RR, [D-Asp^3^]MC-LR, [Dha^7^]MC-LR, MC-(H2)YR, [epoxyAdda^5^]MC-LR, [DMAdda^5^]MC-RR and [Mser^7^]MC-RR	0.2–330 μg/g	[103]
Macrophyte-vegetated lagoons	UHPLC-MS/MS	SPE	-	MC-LR	1.301–11.630 ng/L	[104]
Lake and sea water samples	MSPE (magnetic γ-CDP)-HPLC-MS/MS	MSPE	-	MC-LR, MC-RR and MC-LY	0.8–2.0 pg /mL	[105]
Taihu Lake	CE-ESI-MS	SPE with Sep-Pak C_18_ Cartridge	-	MC-RR, MC-WR, MC-LR and MC-LA	0.2–1 µg/L	[106]
Water samples	ciELISA and LC-MS/MS	-	-	MC-LR and MC-RR	0.02–2.055 µg/L	[107]
Water samples	FELISA	-	-	MC-LR	0.01–2.14 µg/L	[108]
Ter River	UHPLC-HRMS	SPE	-	MC-LR, MC-RR, MC-YR, MC-LA, MC-LY, MC-LW and MC-LF	4–150 pg/L	[109]
Trampling Lake and Pretzlaff Pond	1H and 13C NMR spectroscopy, LC-HRMS/MS and UV spectroscopy	-	*Microcystis aeruginosa*	[D-Leu^1^]MC-LY, [D-Leu^1^]MC-LR, [D-Leu^1^]MC-M(O)R, [D-Leu^1^]MC-MR and [D-Leu^1^]MC-M(O2)R	-	[110]
Amazon River at the Drinking Water Treatment Plant of the Municipality of Macapá, Brazil	ELISA and LC-MS		*Limnothrix planctonica*, *Leptolyngbya* sp., and *Alkalinema pantanalense*	MC-LR	0.026–2.1 µg/L	[111]

### 2.2. Biochemical Method

#### 2.2.1. Protein Phosphatase Inhibition Assay (PPIA)

Microcystins are specific inhibitors of PP1 and PP2A [8,27,31] and thus make PPIA suitable to detect MCs. Since the first establishment of PPIA (by using a colorimetric PPA, which uses substrates such as *p*-nitrophenyl phosphate [112]), various other PPIA techniques have been constructed for MCs detection (Table 1, Table 2, Table 3 and Table 4). A novel colorimetric immune-PPIA (CI-PPIA) where the combination of immune detection and toxicity-based PPI in the CIPPIA provides a useful addition to existing methods [49], colorimetric and fluorometric PPIA, which require an enrichment step using C_18_ cartridges to achieve lower detection limit below the WHO’s provisional guideline value [50]; electrochemical MC-LR biosensor based on the inhibition of recombinant PP1α [70]; and immunocapture PPIA (IC-PPIA), which utilizes antibody to specifically isolate MCs from urine prior to detection through PP2A kit [113] effectively detected different variants of MC. In the inhibition characteristics study of three different protein phosphatases (natural PP2A, recombinant PP2A and recombinant PP1) using three MC variants (MC-RR, MC-LR and MC-YR), MC-LR displayed the highest toxicity followed by MC-YR and MC-RR. The most sensitive enzyme for inhibition by MCs was recombinant PP2A followed by recombinant PP1 and natural PP2A [63]. In a recent study, PP2A inhibition assay using rhPP2Ac was used to detect varying MC variants and toxicities in reservoirs and artificial ponds in Okinawa, Japan, and MC-WR as well as MC-FR were identified for the first time [99]. To quickly assess water and rumen content for MCs, a cost-effective PP1 assay using *p*-nitrophenyl phosphate has been established [81].

Generally, PPIA is merited for being a simple and less expensive technique to monitor MCs. It is also fast and highly sensitive to detect MCs and provides toxicological information to protect human and animal health. For a large number of samples, PPIA is more convenient to use in detecting these deadly toxins [70,81,88,99]. PPIA is also capable of quantifying MCs in water below the WHO’s drinking water guideline level devoid of sample pre-concentration and should be suitable as a regular monitoring technique [68,88,99,114].

The major limitations of PPIA include that it does not provide information on the toxicity of MC variant(s) and that an additional confirmatory method is required for specific analysis due to its lack of specificity for MCs [53,99,114]. Without a cleanup step to isolate MCs from a sample, PPIA cannot differentiate the toxins from other discrete environmental PPI including okadaic acid, calyculin A and tautomyci [49,81]. It is worth noting that complete information concerning chemical characteristics of MCs available in water samples cannot be specified by this technique. Consequently utilizing PPIA as a screening technique will significantly diminish the number of water samples that may need extra analyses [50].

**Table 2:** Analytical methods to detect microcystins in fish/fluids.

Source	Analytical Method (Test)	Sample Preparation	Major Species	MC Variant Detected	Amount of Toxin Detected	Reference
Fish tissue	ELISA and LC-MS/MS	Unbuffered QuEChERS extraction, MeOH, MeCN and d-SPE C_18_ sorbent	-	MC-RR, MC-YR, MC-LR, MC-WR, MC-LA, MC-LY, MC-LW, and MC-LF	14–89 ng/g	[115]
Fish tissue	ELISA and SPME-GC-MS	-	-	MC-LR	0.017–4.69 µg/L	[116]
Fish tissues	LC-MS/MS	-	-	MC-LR	1.0–70 µg/kg	[117]
Human serum	Adda-ELISA (polyclonal antibody) and Adda-ELISA (monoclonal antibody) kit	SPE	-	MC-LR, MC-YR, MC-RR, MC-LA, MC-LW and MC-LF	-	[118]
Finfish and marine mussel tissues	DM-ELISA, anti-Adda ELISA and LC-MS/MS	-	-	MC-RR, MC-LR, MC-LA, MC-WR, MC-YR, MC-LY, MC-LW and MC-LF	-	[119]
Fish from Lake Victoria	ELISA, PPIA and LC-MS/MS	-	*Microcystis, Planktolyngbya* and *Dolichospermum*	MC-LR and MC-YR	-	[120]
Shellfish	HPLC-MS/MS	HLB/PDMS-coated SBSE	-	MC-RR, MCYR, MC-LR, MC-LA, MC-LF, MC-LW and MC-LY	0.1–0.6 μg/kg	[121]
Human urine	LC−MS/MS	-	-	MC-LR	0.500–75.0 ng/mL	[122]
Human urine	IC-PPIA (PP2A)	-	-	MC-RR, MC-LR and MC-LF	0.050–0.500 ng/mL.	[113]
Dog vomitus, blood and urine	Adda-ELISA, LC-MS/MS and MMPB		*Microcystis*	MC-LR, [Dha^7^]MC-LR, MC-HilR, [DAsp^3^]MC-LR, MC-LY, MC-LW and MC-LF	0–14000 ng/g	[123]
Omnivorous crucian carp	HPLC and LC-MS	-	-	MC-RR	0.013–1.592 μg/g dw	[124]
Mice urine, plasma and human serum	UHPLC-QqQ-MS/MS	SPE	-	MC-LR, MC-RR, MC-LA, MC-LF, MC-LW, and MC-YR	0.05–0.30 µg/L	[125]

**Table 3:** Analytical methods to detect microcystins in cyanobacterial cell.

Source	Analytical Method (Test)	Sample Preparation	Major Species	MC Variant Detected	Amount of Toxin Detected	Reference
Algal cultures	CD-ELISA, CI-ELISA and HPLC	-	*Microcystis aeruginosa*	MC-LR, MC-RR and MC-YR	0.10–0.21 ng/mL	[126]
Algae extracts	HPLC-DAD/UV and CE- UV-VIS	SPE C_18_ cartridges and IAC	-	MC-RR, MC-LR and MC-YR	2.12–968.80 µg/L	[52]
Crude algae sample	CZE-ESI-MS	-	-	MC-LR and MC-YR	0.05–0.08 µg/L	[127]
Environmental algal blooms	CE-UV and CE TOF-MS	-	-	MC-RR, MC-LR, MC-YR and MC-LA	0.92–2.3 µg/L	[128]
Cyanobacterial cultures	MALDI-TOF MS, HPLC-DAD and ELISA	MeOH and sonication	*Microcystis aeruginosa*	MC-LR and MC-[D-Asp3]-LR	0.15–0.16 µg/L	[129]
Cyanobacterial cultures	LC-MS/MS		*Microcystis* spp.	MC-LR	0.1–9.1 µg/L	[130]
Environmental samples	ELISA and PPIA	MeOH and C_18_ cartridges	-	MC-LR	-	[131]

**Table 4:** Analytical methods to detect microcystins in dietary supplements.

Source	Analytical Method (Test)	Sample Preparation	Major Species	MC Variant Detected	Amount of Toxin Detected	Reference
*Spirulina* and *A*. *flos-aquae* dietary supplements	LDTD-APCI-HRMS and UHPLC-HESI-HRMS	-	-	MC-RR, MC-YR, MC-LR, MC-LA, MC-LY, MC-LY and MC-LW	0.01–0.3 µg/g	[132]
*Spirulina* dietary supplements	LC-HRMS	-	-	MC-LR, MC-Raba, [Mser ^7^], MC-RY, MC-RY (OMe), MC-LY, [Dha ^7^] MC-LR, [Dha ^7^] MC-YR, MC-LR, MC-RR, and MC-FR	-	[133]
*Aphanizomenon flos-aquae* dietary supplements	PPIA- (PP2A) kit and LC-MS/MS	-	-	MC-LA, MC-LR and MC-LY	≥0.25–2.8 µg/g	[134]
*Aphanizomenon flos-aquae* (Upper Klamath Lake) dietary supplements	HPLC and ELISA	-	*Microcystis aeruginosa*	MC-LR	>1 mg/g	[135]
*Aphanizomenon flos-aquae* dietary supplements	cPPIA, Adda-ELlSA and LC-MS/MS	-	-	MC-LR	≤1 µg MC-LR equivalents g^−l^ dw	[136]
*Aphanizomenon flos-aquae* dietary supplements (capsule, liquid, powder, and tablet)	LC-MS/MS	C_18_ silica- and polymeric-based SPE sorbents	-	MC-LR, MC-LA and MC-LY	0.18–1.87 μg of MC-LR eq/g	[137]
Dietary supplement tablet powder	UHPLC-MS/MS	MeOH	-	MC-LR, MC-RR, MC-LA, MC-LY, MC-LF, MC-LW, MC-YR, MC-WR, [Asp^3^] MC-LR, MC-HilR and MC-HtyR	0.12–1.18 µg/kg	[138]

#### 2.2.2. Enzyme Linked Immunosorbent Assay (ELISA)

Enzyme linked immunosorbent assay, which is capable to detect several MC variants is a good screening technique particularly useful to demonstrate the presence of MCs producing cyanobacteria, track relative changes in MCs concentrations and give clue to control blooms in water source [88]. Owing to the antibodies developed against β-amino acid Adda found in most MC variants, developments of ELISA utilizing polyclonal and monoclonal antibodies have been made possible. The first polyclonal antibody raised against MC was reported by Brooks and Codd [139]; however, its first successful use against MC in rabbit was demonstrated by Chu et al. [140]. Moreover the first use of anti-MC monoclonal was reported by Kfir et al. [141]. To date, several ELISA techniques exist for MCs detection in the environment (Table 1, Table 2, Table 3, Table 4 and Table 5). New ELISA utilizing antibodies extracted from eggs of immunized chickens, competitive indirect ELISA (CI-ELISA) utilizing antibodies raised in sheep against 6(E) Adda, as well as competitive direct ELISA (CD-ELISA) and CI-ELISA generated from rabbits conjugated with gamma-globulin were applied to detected various MC variants in water samples [43,48,126]. Recently, the newly developed CI-ELISA, which utilized MC-LR-keyhole limpet hemocyanin (KLH) for New Zealand white rabbit immunization and produced antibodies, detected MC-LR with a limit of detection (LOD) of 0.0016 ng/mL [107].

To elevate the sensitiveness of ELISA, a modified ELISA described as indirect competitive ELISA (IC-ELISA), which has high antibody specificity for MC arginine in position 4, was generated [56,57]. In subsequent studies, IC-ELISA based on anti-Adda monoclonal antibody (mAb2G5), bifunctional single chain variable fragment-alkaline phosphatase fusion protein (scFv−AP), anti-MC-LR scFv7-scFv (MscFv7-scFv), as well as anti-MC-LR polyclonal antibodies and scFv (PAbs and scFv) were constructed for high MC-LR specificity and sensitivity [79,80,95,98]. It is of interest that the novel fluorometry noncompetitive ELISA based on synthetic broad-specific anti-immunocomplex antibody SA51D1, Fluorescent ELISA (FELISA) based on silane-doped carbon dots and Norwegian ELISA successfully detected varying variants of MC below the WHO’s guideline value of 1 µg/L [87,97,108]. Moreover, to detect MCs in animal cells and tissues, a direct monoclonal ELISA (DM-ELISA) has been developed for rapid and easy detection [119].

ELISA is merited for being highly specific, sensitive, and quick to perform. It is very useful for first examination and rapid to detect MCs. A small amount of water sample is needed for toxins identification. Generally, ELISA is capable of yielding repeatability, reproducibility and variability results of MCs concentrations compared to the other methods [86,87,107,118]. Besides, no sample cleanup is needed, detection limits are often below the WHO’s 1 µg/L guideline value, and it is sensitive to low pH (formic acid), MeOH or MeCN [79,87,88,114,115]. ELISA can be used to determine the biological evidence of human exposure to MCs. The Adda-ELISA (polyclonal and monoclonal antibody) kit for serum (Serum-ELISA) is an appropriate technique for preliminary screening and serves as a suitable technique to analyze MCs in human blood serum in a cost effective manner [26,114,118]. ELISA can also be used to detect MCs in various animal cells or tissues as the toxin can accumulate in seafood [86,93,119]. Further, this technique is capable of detecting MC covalent bound. Through the residue of Mdha, MC may form covalent bonds with the catalytic subunits of PP1 and PP2A, which are mainly found in liver tissue [81,82]. It is worth knowing that ELISA kits are easy to operate on the field and offer a simple monitoring technique, which immediately detects MCs. In addition, the dipstick format of ELISA kits allows a quick screening to detect MCs in raw or treated water. To screen several samples at once, ELISA kits are more appropriate due to the configuration in the 96-well plates [97,114], though plate readers for ELISA are moderately expensive, especially in comparison to mass spectrometers or HPLC equipment.

ELISA is restrained by expensive equipment cost and involves a relatively long procedure in its analysis and interpretation. This makes it time consuming and also requires trained personnel to operate. Though ELISA is sensitive, it is not suitable to be used as standard analytical technique to detect MCs. This is because it does not directly measure MC, frequently distinguishes MC variants (this is because they recognize the Adda moiety that is present in almost all MC variants), is unable to specify the relative toxicity of MC variants and bases its calibration curves on log or semi-log plots [26,86,97,115]. The accuracy of this technique depends on matching the calibration standards to the variant(s) being measured in samples (example MC-LW standards for measuring MC-LW); therefore, if the calibration standards are not matched to the variant(s) being measured in the samples, the measured concentrations may appear to be much higher than actual concentrations, depending on the variant(s). ELISA has a limited quantification range (0.15–5 µg/L), which suggests that higher concentration samples must be diluted [88]. Hence, this technique should be used with caution for absolute quantification, particularly at high concentrations. Interestingly an ELISA antibody developed against specific MC variant(s) may give false negatives if used to detect other MC variant(s) to which it is not sensitive to. The exact MC content and toxicity are likely to not be detected due to the variations in the specificity of the antibody [43,48,107,126]. Even though ELISA is capable of detecting MCs in solid samples (including fish tissues), the extraction methods it uses (for the solid samples) are not typically suitable for field application (non-solid samples) [82,115,119]. Moreover, its ability to analyze MCs in human blood serum is hindered by the time needed for sample preparation or overestimation of some specific MC variants concentrations [118]. ELISA kits lack the ability to differentiate between MC variants for quantitative purposes, and the dipstick can be difficult to read [86,114,115]. In addition, incidents of false positives in ELISA are more feasible compared to false negatives, which are not persistent, and are somewhat compromised by matrix effects [88,114,119]. In view of this assertion, quality control has become obligatory and can be achieved through spiking samples with known amounts of MCs and confirming them with other methodologies.

### 2.3. Chemical Method

#### 2.3.1. High Performance Liquid Chromatography (HPLC)

The most commonly and widely used laboratory technique to analyze MCs by means of different stationary and aqueous mobile phases containing methanol or acetonitrile is HPLC and its linked techniques (Table 1, Table 2, Table 3, Table 4 and Table 5). Ultraviolet-visible spectroscopy (UV–Vis) absorbance and photo-diode array (PDA) detection techniques are mostly associated with HPLC system. Generally, MCs have UV absorption between 190 nm and 300 nm, with a maximum at 238 nm. The most commonly used detection is UV absorption at 238 nm, which is usually performed with PDA detectors. However, MC variants that contain tryptophan indicate a maximum absorption at lower wavelengths of 222 nm [42]. To obtain adequate resolution for MCs detection, HPLC relies on the use of high-resolution RP C_18_ columns, 15 or 25 cm in length and 3 to 5 mm in width. It is worth noting that the confine range of MCs detection is associated with concentration factors attained and the volume of sample. Parameters such as mobile phase composition and HPLC conditions including flow rate, temperature and column features (including stationary phase, silanol activity and length) may account for an excellent separation and sensitivity of HPLC [69,92,100]. For successful use of this technique, a worldwide certified reference material to purify and quantify MCs has been acknowledged. This will help to ensure standardization of routine laboratory analysis of these toxins.

To improve upon the sensitivity and selectivity of HPLC, novels including magnetic solid-phase extraction (MSPE) coupled with HPLC/UV based on a magnetic bentonite sorbent fabricated by solvothermal synthesis method, MSPE based on mesoporous Fe3O4@mSiO2@Cu2^+^ nanoparticles (NPs) coupled with HPLC, and MSPE coupled with HPLC/UV where the magnetic composite material was combined with cetylpyridinium chloride prepared by hydrothermal synthesis [71,72,89] were developed and validated for trace detection and analysis of MCs.

The use of HPLC is generally associated with the following merits. To confirm and identify MC variants in an unknown sample, HPLC is preferred since it provides enough information on the MC variant(s) present. HPLC can also be used to generate both quantitative and qualitative data for MCs analysis [35,72,89]. In addition, it is capable of identifying and quantifying MC variants in a sample if suitable analytical standards are present. For UV detectors, LOD to determine MCs is below 1 µg/L, which is suitable to detect trace amounts of MCs in water samples [71,76,91]. It is worth noting that individual MC are capable of being separated and recognized when MC standards are matched by their retention time (RT), and characteristic UV absorption spectra (kmax) [39,69,74]. Further, the PDA detector records the spectrum in addition to the UV response of the analyte, which to a larger extent gives better proof of the presence of MCs. The mobile phase also has the ability to detect MCs that are resolved from each other [35,64,74,82]. It is of interest that this technique allows for the accurate detection of both intracellular and extracellular toxins of MCs [42,64,77,90].

HPLC is constrained by being technically demanding, expensive, time consuming and requires an expert in the field to operate as well as extensive sample cleanup. MCs detection by HPLC depends on on-site sampling, sample processing and laboratory analysis which can be time demanding. Moreover, due to its low selectivity and time response, it is not suitable for rapid processing of multiple samples [75,77,94,100,129]. HPLC is also constrained by the large number of diverse MC variants and the commercial availability of standard compounds without which identification of MC becomes impossible. Further, due to the slight difference that exists between MC variants, it sometimes becomes very difficult to use this technique for separation [33,89,90,91]. Interestingly, HPLC cannot differentiate between structural MC variants, and the retention time may not be an appropriate explicit detector for the toxins. This is because similar structures of MC are capable to co-elute. Besides, by quantifying MC peaks, difficulties may be caused via the appearance of additional peaks in the HPLC chromatograms as a result of leaching of material from the C_18_ trifunctional (C_18t_) SPE cartridges, and co-elution of other organic compounds in water sample. This makes it difficult to identify MCs using their characteristic UV spectra, especially at low toxin concentrations [35,38,42,69,91].

#### 2.3.2. Liquid Chromatography-Mass Spectrometry (LC-MS)

This is a sophisticated technique to detect various variants of MC in the environment. MS detection, which acts as a fingerprint, depends on the availability of respective MC variants’ standards and the levels of non-covalently bound MCs in the sample. In HPLC systems with MS detection, shorter columns (3–10 cm) are usually used [81,86,100]. In the late 1950s, the first attempt to detect the structure of MC was made [147]; however, full structural detection was achieved with the application of MS in 1984 [148]. An important expansion of the MS detection is the MS/MS detection, where the fragmentation model can be greatly used to assist in the identification of unknown MCs [34,88,96,142]. At present, a number of LC-MS techniques exist for MCs determination (Table 1, Table 2, Table 3, Table 4 and Table 5).

Mass spectrometry novels including double-sided magnetic molecularly imprinted polymer modified graphene oxide (DS-MMIP@GO) based MSPE combined with LC-MS/MS (DS-MMIP@GO and LC–MS/MS), a new approach based on molecular networking analysis of LC-MS/MS, time-efficient LC-MS precursor ion screening method that facilitates MCs detection and identification, and immunocapture LC−MS/MS using an antibody that recognizes the Adda portion of MCs [26,73,75,122] have been established and validated for effective MCs identification. Besides, the developed paper spray ionization method coupled with a time of flight (TOF) MS and a filter-feeder organism coupled with matrix-assisted laser desorption ionization-TOF MS (MALDI-TOF MS) were found to yield effective MCs determination in various water samples [83,129]. It is worth knowing that the high throughput method based on on-line SPE coupled to LC–quadrupole TOF resolution MS (on-line SPE-LC-QToF HRMS) (LOD between 0.004 and 0.01 µg/L), on-line SPE-UHPLC-HRMS (LOD between 8 and 53 ng/L), online SPE-UHPLC-MS/MS (LOD between 0.1 and 0.5 µg/kg) and online concentration LC/MS/MS workflow (LOD between 0.6 and 3.8 ng/L) also successfully detected different variants of MC in water samples [34,96,101,102]. The matrix solid-phase dispersion (MSPD) followed by HPLC/MS/MS (MSPD-HPLC-MS/MS) (LOD 13.0 μg/kg (dw)) and SPE-UPLC-MS/MS (LOD 0.06–0.42 ng/g f.w) were also used to determine trace levels of MCs in various vegetables [142]. To detect and quantify MCs in animal tissue LC coupled with tandem quadrupole MS (LC-MS/MS), and hydrophile lipophile balance/polydimethylsiloxane (HLB/PDMS)-coated stir bar sorptive extraction (SBSE) coupled with HPLC-MS/MS have been developed [117,121].

Generally, LC-MS is merited for being capable to provide efficient exposition for MCs structure. Although LC/MS detectors are limited by equipment cost, they are becoming cheaper, and thus, a water monitoring laboratory with a limited budget may soon be able to purchase them for routine analysis. It is of interest that MS can be used to separate, quantify and present potential for high throughput towards MCs detection [101,102,103,122]. With the availability of suitable analytical standards, the technique can be used to confirm, identify and quantify variants of MC in an unknown sample, and regarding method specificity LC/TOF-MS is preferred due to its accurate mass capability [83,84,88,103,129]. This may provide the specificity and sensitivity needed to advise operational decisions for MC variants found in drinking water sources. Further LC-MS is capable to detect MCs in blue-green algae (BGA) dietary supplements, vegetables, animal cell or muscle tissue and human serum [96,121,122,126,132,142]. This puts much emphasis on the significance of examining MCs in dietary supplements that people consume due to the health benefits derived, vegetables, and rivers as well as fishponds that serve as sources of fish for human consumption. Since MSPD-HPLC-MS/MS has as characteristics using smaller sample sizes, lower costs and wider applicability in analytical laboratories, it is considered appropriate and can be widely adopted in the field of food safety and control. This can facilitate further research about the spatial and temporal distribution of MCs between water samples, vegetables and human health risks due to the explosion to MCs through edible vegetables in the future [142]. In addition, for MCs detection in unusual matrices such as benthic biofilms or lichen, LC-MS precursor ion screening method is considered useful [26].

LC-MS limitations include requiring personnel with specialized training to operate. Moreover, to analyze and interpret LC-MS results, more time is needed to perform this operation. For MS sample enrichment and cleaning, complex preparation is needed due to its level of sensitivity and selectivity [34,93,120]. Further, though MS/MS detection is more sensitive and selective, standard reference is needed to detect the optimal ion transitions of the analyses and for quantitative purposes [101,102,103,115].

#### 2.3.3. High Performance Capillary Electrophoresis (HPCE)

This technique is considered for the separation and quantification of MC variants mostly in relation to their differences in molecular size and charge. The analysis of this technique is short, with high separation efficiency, uses small sample volume, low solvent cost and is also associated with little hazardous waste [44,106,128]. HPCE has successfully been applied to analyze MCs in crude algae samples since its development (Table 1; Table 3). CE method incorporating sodium dodecyl sulphate (SDS)-organic modifier solvents, capillary electro-chromatography (CEC) in reversed-phase capillary formed by inorganic or organic polymer monoliths, home-made monolithic columns in high pressurized CEC-UV (pCEC-UV), capillary zone electrophoresis (CZE) and micellar electrokinetic chromatography (MEKC) coupled with UV, and CZE coupled with electrospray ionization mass spectrometry (ESI-MS) (CZE-ESI-MS) [45,55,58,60,127] are some of the techniques developed for MCs determination, separation and quantification. In recent investigations, the developed CE TOF-MS (LOD between 0.92 and 2.3 µg/L) and dynamic pH junction in CE-ESI-MS based on electrophoretic mobility difference of analytes in sample matrix and the background electrolyte (LOD between 0.2 and 1 µg/L) successfully demonstrated separation and identification of different MC variants commonly found in aquatic environments [106,128].

HPCE is merited for being fast and easily applicable for the separation of MCs with similar charge-to-mass ratio. High-efficiency separation of toxic peptide molecules having equal or nearly equal mass to charge ratios can be achieved using SDS as an additive to the running buffer. Interestingly, the migration time, elution order, baseline separation and selectivity of MCs using the CE system by modifying the composition of the buffer with organic solvents can be influenced [45,52,106,127]. Further, CE has the ability to separate isomers differing only in the position of a single methyl group on MC structure [44,128]. It is worthwhile noting that the sensitivity of CE has been elevated by improving sample pretreatment protocols and electrophoretic conditions. Utilizing immunoaffinity chromatography (IAC) for sample pretreatment and on-line pre-concentration approaches such as field-amplified sample stacking (FASS) for the concentration of sample that allows MCs to be detected at trace level, the sensitivity of CE has improved. The selectivity has also been increased via MEKC mode in the CE analysis [51,52,128].

HPCE is, however, still limited by sensitivity. Comparing to HPLC procedure, CE has low sensitivity, making it not suitable or robust for routine monitoring of water sample at the laboratory. Although the sensitivity of HPCE approach has been improved through IAC, FASS and dynamic pH junction [51,52,106], further developments are still needed to increase the flow cell volume to improve the detection limit.

#### 2.3.4. Gas Chromatography (GC)

GC has been in existence since the early 1990s to detect MCs (Table 1; Table 2). This technique is based on the oxidation of MCs which splits the Adda side chain to produce 2-methyl-3-methoxy-4-phenylbutyric acid (MMPB). Producing MMPB requires treating MCs with ozone [46] and also oxidation of MCs via permanganate and periodate [47]. MC variants that contain Adda give rise to one MMPB molecule and are therefore detected with equal sensitivity. Interestingly, GC/MS can also be applied to detect MMPB [47,85,116].

GC/chemical ionization MS (GC/CI-MS) method, an improved GC using ozonolysis where direct intact analysis of MMPB are done by thermospray (TSP) LC/MS (TSP-LC/MS) and electron ionization GC/MS (EI-GC/MS), an improved recovery MMPB technique specifically the use of SPME as a unique and novel application in GC-MS (SPME-GC/MS) and GC-MS method based on the derivatization of MMPB with methylchloroformate (MCF) [46,47,67,116] have been generated to screen and quantify MCs in blooms and cultured samples. The recently developed novel including ozonolysis, MMPB with MCF and GC-MS (LOD 0.34 µg/L) was found to detect varying MC variants in water samples [85].

GC is generally merited for the following. The total MCs can be evaluated without knowing the MC variant(s) present in the sample and without determining each component. This makes it a usable alternative for environmental safety and health concerns caused by the existence of MCs in CyanoHABs [85,116]. GC is accurate as it is able to measure MMPB resulting from Adda and also has the potential to detect other Adda-containing toxins in water. Moreover, in terms of quantitative analysis, it does not require MCs standards to detect these toxins [46,47,67,85]. MMPB is also capable of detecting the concentration of free and covalently bound MCs in a sample. It can also be applied to detecting MCs in complex samples such as sediments and animal cells or tissues [149]. The SPME-GC/MS is an effective and efficient technique to measure total MCs content in biological matrices such as animal tissues. This, therefore, represents a powerful means to enhance our ability to understand the bioaccumulation of these toxins including freshwater, food webs, assessment of human exposure and its potential implications on human health [116]. Further MCs can be oxidized by ozone to produce MMPB at ambient temperature. This suggests that ozonation may be an effective and rapid method for the transformation of MCs to MMPB without secondary pollution [85].

Limitations of GC including less sensitivity, time consuming and unable to differentiate between different MC variants have caused restriction in its usage. Since individual MC cannot be detected, GC cannot therefore be used to monitor water samples with regards to the proposed water guideline by the WHO. In addition, GC involves some tiresome measures throughout the removal, cleanup, oxidation and post-treatment in order to get rid of the reagents used and derivatization for its analysis [46,67,85].

## 3. Biosensor Methods to Detect Microcystins

To date, the methods described in Section 2 have been employed to monitor and detect different MC variants in the environment. While these techniques are precise and sensitive, expensive instrumentation, well-trained personnel and time-consuming procedures are involved. This suggests that their applications may primarily be limited to well-resourced and centralized laboratory facilities. Consequently, development of low-cost and ultrasensitive measuring method would help limit exposure by enabling early detection and continuous monitoring of MCs.

In recent times, the robust, simple, specific, sensitive, portable, easy to utilize and rapid biosensor method which functions as an enhanced monitoring tool for MCs, particularly to analyze low MCs concentrations and manage the risk associated with health, is gradually gaining global focus. Biosensor is an analytical tool made up of a biological recognition element termed as bioreceptor, in direct contact with a transducer. This analytical tool can be classified either by their biological recognition element or signal transduction methods. A bioreceptor in biosensor is often combined with a suitable transduction method to generate a signal following interaction with the target molecule of interest [150,151,152,153]. It is worthwhile noting that various natural and artificial biological elements including whole cells, enzymes, antibodies, molecularly imprinted polymers (MIPs) and nucleic acids are employed in biosensors [151,153,154,155].

At present, enzyme based biosensors (including optical and electrochemical biosensors), immunosensors (including electrochemical, piezoelectric, NMR-based and optical immunosensors such as Surface Plasmon Resonance (SPR) immunosensors, Evanescent Wave Fiber-optic immunosensors, Luminescent immunosensors, Fluorescent immunosensors, and Immunoarray biosensors) and nucleic acid biosensors (including electrochemical DNA and SPR-DNA biosensor) have been developed for successful MCs determination [151,152,153,156]. A rapid and sensitive SPR biosensor, which incorporates commercial Adda-group antibody (Ab) and has the capacity for broader recognition of various MC variants than earlier developed sensors for BGA supplements was established and validated. This technique is capable to further observe BGA products to aid risk assessment, ascertain regulatory guidance levels and respond to potential consumer complaints linked to BGA products [155]. The constructed electrochemical biosensor that possesses good stability against other components in natural water sample, prepared via physically immobilizing calf thymus DNA (ctDNA) on gold electrode after characterizing the deleterious effect of MC-LR to ctDNA, was utilized to detect MC-LR in local water bodies. The technique indicated linear range of 4–512 ng/L, LOD of 1.4 ng/L (perceived to be 700-fold lower than WHO’s suggested guideline value) and recoveries were 95.1% to 107.6% [156]. A novel fiber optical chemiluminescent biosensor (FOCB) system was successfully generated utilizing fiber optic bio-probe as biorecognition element as well as transducer and a Si-based photodiode detector (PD-3000). The FOCB system is robust, portable, cost-effective, utilizes small sample volume, is suitable for on-site and automatically detects targets. A highly sensitive MC-LR determination was attained with LOD of 0.03 µg/L under optimal conditions and recoveries were in the range of 80% to 120% [150]. A Surface-Enhanced Raman Scattering (SERS) spectroscopic immunosensor with outstanding sensitivity, selectivity and robustness was established to detect and quantify MC-LR in aquatic settings. The established SERS sensor could reach LOD (0.014 µg/L) at least 1 order of magnitude lower, exhibited a linear dynamic detection range (0.01 µg/L to 100 µg/L) 2 orders of magnitude wider in comparison to the conventional techniques, and recoveries were 100% to 107%. Further, the SERS immunosensor enabled monitoring of the dynamic production of MC-LR from a *Microcystis aeruginosa* culture [154]. The developed phosphorescent immunosensor, which acquired antigens and antibodies as recognition units and employed Mn-ZnS RTP QDs as sensing materials to specifically bind with MC-LR, also demonstrated rapid and sensitive MC-LR detection with linear ranges of 0.2–1.5 µg/L and 1.5–20 µg/L, LOD up to 0.024 µg/L, and recoveries were 93.1% to 105%. Interestingly, no significant obstruction was observed from coexisting MC-LR pollutants in water during the toxin’s determination [152]. Further, a novel Cu/Au/Pt trimetallic nanoparticles (Cu/Au/Pt TNs)-encapsulated DNA hydrogel prepared for colorimetric detection of MC-LR also detected the toxin with a linear range of 4.0–10.000 ng/L, LOD of 3.0 ng/L, and recoveries of fresh crucian carp tissue were in the range of 95.34% to 107.07% while the recoveries of water ranged from 93.96% to 105.33% [157].

Aptasensors are biosensors that employ aptamer as recognition element. In developing aptasensors, nanomaterials that are regarded as potential agents are mostly considered because of their physico-chemical properties including small size, disposability and high surface area [158]. Various highly specific and sensitive aptasensors-based optical (such as colorimetric, fluorescent, SERS, Electrochemiluminescence (ECL) aptasensors) and electrochemical-based aptasensors currently exist for the determination of MC-LR [158,159,160]. A novel aptasensor based on SERS where MC-LR aptamer and its corresponding complementary DNA fragments (cDNA) were conjugated to gold nanoparticles (AuNPs) and magnetic nanoparticles (MNPs), respectively, used as signal and capture probes (aptamer-AuNPs and cDNA-MNPs conjugates) was constructed and applied for highly sensitive MC-LR detection. The technique revealed a linear range from 0.01 to 200 ng/mL, LOD of 0.002 ng/mL, and the recovery values ranged from 88.84% to 105.72% [160]. A sensitive and selective electrochemical aptasensor that exhibited a linear range of 0.005–30 nM, LOD of 0.002 nM and recovery rates from 95% to 106% for MC-LR determination was developed based on a dual signal amplification system comprising of a novel ternary composite (prepared via depositing AuNPs on molybdenum disulfide (MoS_2_) covered TiO_2_ nanobeads) and horseradish peroxidase (HRP) [159]. Further, a novel dual-mode aptasensor based on MoS_2_-PtPd NPs and zeolitic imidazolate framework (ZIF)-8-thionine (Thi)-Au (ZIF-8-Thi-Au) (as signal material) was established and demonstrated ultra-sensitive and quick MC-LR detection. The aptasensor indicated a liner range from 0.01 to 50 ng/mL, lowest LOD at 0.006 ng/mL, and recovery was from 95.5% to 109.6% [161]. The collective effects of these methods were evaluated using the recovery rate. The findings demonstrate that the biosensors recoveries ranged from 88.84% to 109%. The good recovery rates exhibited indicate that the biosensors possess good stability against other components (matrix effect) in water and fish samples.

The ability to assess health status, disease onset and progression, and monitor treatment outcome is the primarily objective in health care promotion and delivery. Biosensors and point-of-care devices have the potential to improve delivery of healthcare. The latest development in biosensor technologies can deliver point-of-care diagnostics that match or exceed conventional standards in terms of cost, time and accuracy [162,163,164]. However, the practical application of biosensors in medical diagnosis and treatment is still advancing. Since the development of the first glucose electrochemical sensor, substantial efforts to construct implantable biosensors have been made. Although the devices may be challenged with matrix effects and sample preparation, they can be used to monitor patients, improve the management of patient health and quality of life, enable drug treatments to be administered at specified times, increase survival rates and reduce health care costs and the number of invasive interventions required [162,163,164]. A precise diagnostic for a disease is essential for a successful treatment and recovery of patients suffering from it. Diagnostics methods must be simple, sensitive, detect multiple biomarkers, perform multiplex analysis and assimilate different functions. With successful biosensor integration, biomarkers can be monitored in samples such as saliva, sputum, blood, stool, swab, skin and interstitial fluid [162,163,164]. The electrochemical and optic based biosensors are mainly used for routine evaluation of blood parameters like urea, creatinine, glucose and lactate, as well as point-of-care testing of glucose in clinical chemistry laboratories. Moreover, for high sensitivity and faster analysis in near-patient testing for cardiac and few cancer markers, immunosensors are preferred [162,165]. Most studies concerning MCs detection have been based on water and biological samples. Although biosensors have been used to detect MC-LR, their application in the context of medical diagnosis and the associated matrix effects regarding MC intoxication are yet to be determined. Further studies are therefore recommended. 

It is of interest that biosensors are considered as catalytic (enzymes and whole cells) or affinity (antibodies and nucleic acids) based on their biological elements. The presence of this biological element makes biosensor system very specific and highly sensitive. This gives an upper edge over the conventional methods and bioassay in environmental sensing and detection [151,152,153]. Moreover, an ideal biosensor incorporates features of minimal training, power requirements, portability and presents meaningful results using less sample volumes and reagents. Biosensors can achieve low detection limits of MC-LR in dietary supplements as well as various aquatic settings such as drinking water, lakes, and reservoirs due to the selective binding or reaction of the biological recognition element to the target analyte. The technique can also demonstrate good recovery, precision, and accuracy through the evaluation of the spiked water samples and can be readily extended toward the on-site real-time sensitive detection of other targets in the field of environment, food and medical diagnosis [150,153,154,155]. It is worth-knowing that the aptamers can easily be labeled and fabricated into diverse aptasensors to acquire rapid, sensitive, and specific MC-LR detection. Aptamers demonstrate high affinity, and most of the developed aptasensors are simple to perform with miniaturized instruments to attain on-site monitoring of the toxins. Aptamers also show significant advantages in terms of low generation cost, low molecular weight and quick chemical synthesis and modification. Moreover, they can offer rapid and accurate determination of MC-LR and can be referred to detect other hazardous substances in water products [159,160,161].

## 4. Conclusions

Presence of MCs threatening humans, animals and plants, and the many problems associated with these toxins have called for water attention awareness in many countries across the globe. Constant monitoring for MCs in drinking water, recreational water and other potential avenues has become vital in order to effectively manage and control MCs and prevent or minimize the health risks associated with the toxins’ pollution. For better monitoring, sensitive, fast and reliable screening methods capable of detecting MCs in the environment are urgently required at an early stage. In this paper, the analytical methods to detect MCs ranging from biological (MBA), biochemical (PPIA and ELISA) and chemical (HPLC, LC-MS, HPCE and GC) as well as the newly emerging biosensor methods were reviewed in terms of their novel development, usage, merits and limitations.

Mouse bioassay is useful for initial screening of MCs in samples of unknown toxicity, and it makes effective use of the whole animal. However, it lacks a realistic way to analyze MCs, gives poor quantitative data, and for ethical reasons is very seldom used for testing if at all. PPIA is highly sensitive to detect and quantify MCs and provides toxicological information to protect human and animal health. Nevertheless, it is not specific and does not provide information on the toxicity of MC variants therefore requiring additional confirmatory for specific analysis. The ELISA technique is useful for routine screening of water and capable of detecting the total amount of MCs due to its high sensitivity and specificity. Nonetheless, it is unable to distinguish MC variants and relative toxicity and may strongly be affected by matrix effects. The most reliable technique is HPLC-based methods where standards for the toxins present are available and LC-MS for confirmation, identification and quantification of MC variants mainly in the laboratory. HPLC and HPLC-MS are effective and powerful techniques to detect MCs in complex matrixes, although methods based on HPLC alone fail to provide structural information on MCs. Moreover, many of the classic analytical methods usually need complex sample pretreatment to remove the reagents used and derivatization for HPLC analysis. To combat the various limitations, these three methods should be made relatively affordable to be purchased and used. Moreover, further extensive research aimed at improving these methods for better use is required, especially for field applications to detect MCs in the future. It is of interest that much attention should also be given to the emerging biosensors because of their remarkable sensitivity, selectivity, simplicity and portability. The development of biosensors offers rapid and accurate detection, as well as high reproducibility of MC-LR. Besides, the satisfactory recoveries of these methods signify that they possess good accuracy, respond quickly and avoid interference; therefore, their application for MCs detection should be encouraged. However, further investigations are required to determine the other MC variants in water and biological samples using the biosensor method.

## Figures and Tables

**Table 5:** Analytical methods to detect microcystins in vegetables.

Source	Analytical Method (Test)	Sample Preparation	Major Species	MC Variant Detected	Amount of Toxin Detected	Reference
Vegetables (tomato, cucumber and spinach)	HPLC-MS/MS	MSPD	-	MC-LR, MC-RR, MC-YR, MC-LW and MC-LF	1.5–13.0 mg/kg dw	[142]
Lettuce	UPLC-MS/MS	SPE	-	MC-LR, MC-RR and MC-YR	0.06–0.42 ng/g f.w	[143]
Leaves and roots of vegetable plants	ELISA and HPLC	-	*Oscillatoria limnetica*	MC-LR	0.07–1.2 µg/L f.w	[144]
Carrot	ELISA	-	*Microcystis aeruginosa*	MC-LR	0.47–5.23 ng MC eq./g f.w	[145]
Leaves and fruits of *Capsicum annuum*	HPLC	MeOH and sonication cartridges (OASIS HLB Cartridge)	-	MC-LR and dmMC-LR	-	[146]
